# HSP60-regulated Mitochondrial Proteostasis and Protein Translation Promote Tumor Growth of Ovarian Cancer

**DOI:** 10.1038/s41598-019-48992-7

**Published:** 2019-09-02

**Authors:** Jianying Guo, Xiao Li, Wenhao Zhang, Yuling Chen, Songbiao Zhu, Liang Chen, Renhua Xu, Yang Lv, Di Wu, Mingzhou Guo, Xiaohui Liu, Weiguo Lu, Haiteng Deng

**Affiliations:** 1 MOE Key Laboratory of Bioinformatics, School of Life Sciences, Tsinghua University, Beijing, 100084 China; 2 Department of Gynecologic Oncology, Women’s Hospital, Zhejiang University School of Medicine, No.1 Xueshi Road, Hangzhou Zhejiang, 310006 China; 3 School of Nursing, Binzhou Medical University, Yantai, 264003 China; 4 Department of Gastroenterology and Hepatology, and Center of Nephrology, Chinese PLA General Hospital, Beijing, China; 5 State Key Laboratory of Cardiovascular Disease, Fuwai Hospital, Beijing, 100037 PR China

**Keywords:** Cancer, Biomarkers

## Abstract

Ovarian cancer (OC) is the most lethal gynecological carcinoma due to the lack of diagnostic markers and effective drug targets. Discovery of new therapeutic targets in OC to improve the treatment outcome is urgently needed. We performed proteomic analysis of OC specimens and the paired normal tissues and revealed that proteins associated with mitochondrial proteostasis and protein translation were highly expressed in ovarian tumor tissues, indicating that mitochondria are required for tumor progression of OC. Heat shock protein 60 (HSP60), an important mitochondrial chaperone, was upregulated in ovarian tumors. HSP60 silencing significantly attenuated growth of OC cells in both cells and mice xenografts. Proteomic analysis revealed that HSP60 silencing downregulated proteins involved in mitochondrial functions and protein synthesis. Metabolomic analysis revealed that HSP60 silencing resulted in a more than 100-fold increase in cellular adenine levels, leading to increased adenosine monophosphate and an activated AMPK pathway, and consequently reduced mTORC1-mediated S6K and 4EBP1 phosphorylation to inhibit protein synthesis that suppressed the proliferation of OC cells. These results suggest that HSP60 knockdown breaks mitochondrial proteostasis, and inactivates the mTOR pathway to inhibit OC progression, suggesting that HSP60 is a potential therapeutic target for OC treatment.

## Introduction

Ovarian cancer (OC) is the most deadly gynecological carcinoma and is the eighth leading cause for cancer mortality in women around the world^1^. It is difficult to diagnose epithelial ovarian cancer (EOC) at the early stage owing to the deep location of ovaries in the pelvis, and approximately 60% of patients are diagnosed at the advanced stages^2^. Surgical debulking and platinum-based chemotherapy or radiotherapy are standard therapies for EOC^3^. Despite of improved treatment strategies for EOC, the 5-year survival rate of advanced EOC patients is rather low at less than 30%^4^. Patients with recurrent EOC after initial therapy have few treatment options that greatly compromises their quality of life and life expectancy. Discovery of new therapeutic targets in EOC to improve the treatment outcome is urgently needed. Proteomic analysis is one of the approaches to identify therapeutic targets in OC. Proteomic analysis was applied to 174 ovarian tumors previously analyzed by The Cancer Genome Atlas (TCGA) and identified a strong association between specific histone acetylation and the homologous recombination deficiency phenotype^5^. A recent study has also applied multi-level proteomics to platinum-sensitive and -resistant high-grade serous ovarian cancer (HGSOC) patients, in which CT45 was recognized as a therapeutic target in OC^6^. However, because of the difficulty in collection of normal ovarian tissues from the same ovarian cancer patients, few proteomic studies have been performed to identify differentially expressed proteins between ovarian tumors and the paired normal ovarian tissues, which has limited our understanding of crucial proteins and pathways altered in ovarian tumors. In the present study, we characterized the proteomes of paired ovarian tumors and normal tissues of the same patients to find proteins and signaling pathways that may be therapeutically valuable for OC treatment. Additionally, we determined whether heat shock protein 60 (HSP60) was essential for OC cell progression.

HSP60 is a mitochondrial chaperone that maintains mitochondrial proteostasis. It plays a key role in the folding and transport of mitochondrial proteins and is associated with various types of cancer^7^. HSP60 silencing makes cytochrome *c* release from mitochondria, which causes caspase-dependent death of tumor cells, suggesting that HSP60 is anti-apoptotic in tumors^8^. However, HSP60 also plays a pro-apoptotic role by facilitating activation of pro-caspase 3^9^. Notably, HSP60 is either significantly elevated or decreased in a number of cancers^10^. Our earlier studies demonstrated that HSP60 knockdown interrupted the integrity of respiratory complex I, leading to reactive oxygen species (ROS) overproduction to activate the AMPK pathway, which drove cell growth of clear cell renal cell carcinoma (ccRCC). ccRCC exhibits the classic Warburg phenotype with increased glycolysis and dysfunctional mitochondria^11^. We further showed that activated AMPK inhibits the mTOR pathway to suppress glioblastoma growth^12^. This leads to our central hypothesis that HSP60 plays distinct roles in regulating tumorigenesis and progression of various tumors, because HSP60 promotes tumor progression of glioblastoma (GBM) but inhibits tumor progression of ccRCC. Therefore, it is important to investigate the effects of HSP60 expression on OC progression.

Some studies have reported that higher level of HSP60 expression in ovarian tumors correlated to the shorter overall survival^13^. These findings indicate that HSP60 is a potential target for OC treatment. In the present study, we analyzed HSP60 expression in ovarian tumors and examined its effects on tumor progression. We found that HSP60 was highly expressed in ovarian tumors, and knockdown of HSP60 significantly impeded cell proliferation by disrupting mitochondrial proteostasis and activating the adenine-dependent AMPK pathway, indicating that HSP60 is a potential target for OC therapy.

## Results

### Proteins associated with oxidative phosphorylation and protein translation are upregulated in ovarian tumors compared with the normal ovarian tissues

**Figure 1:**
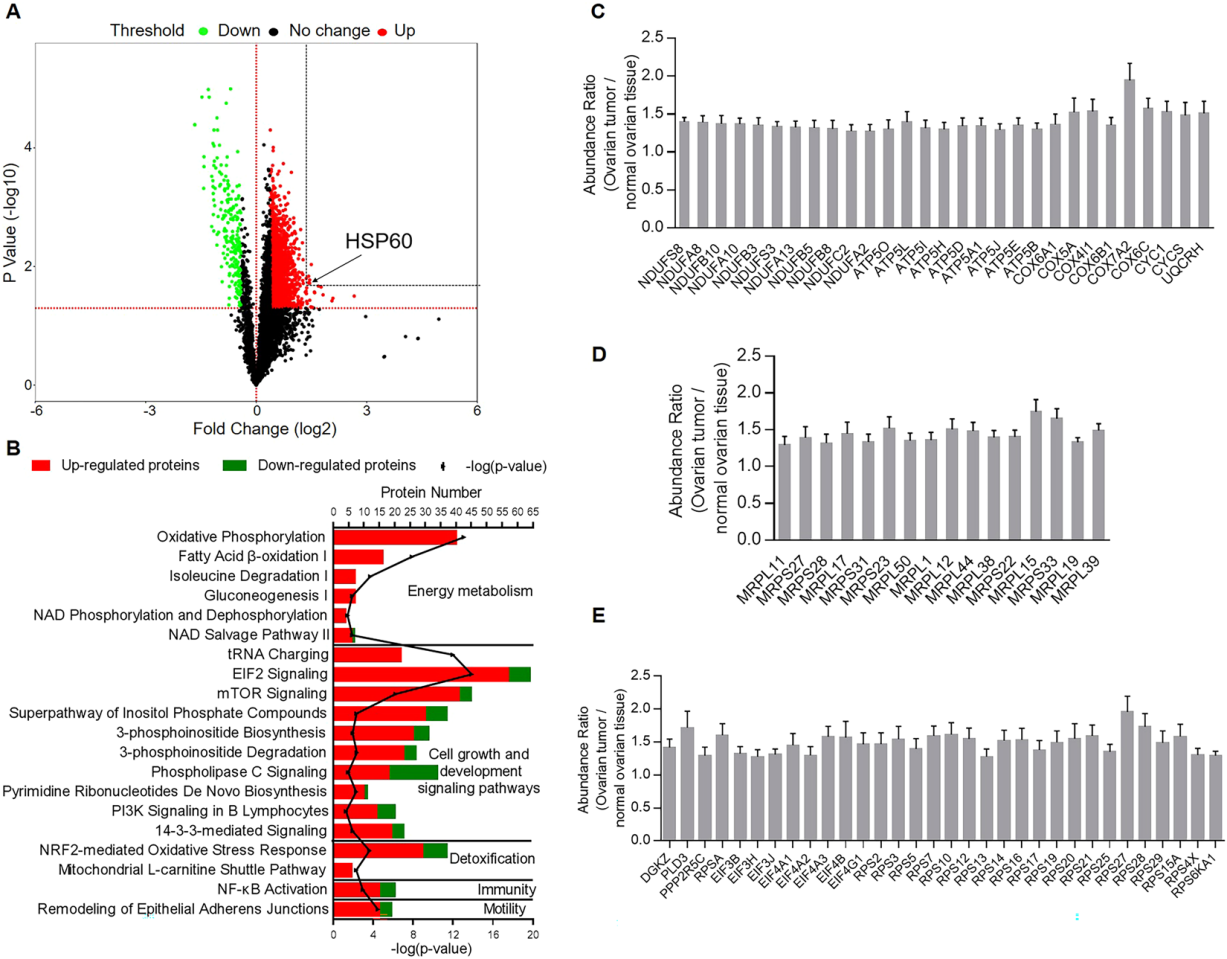
Proteins related to oxidative phosphorylation and protein synthesis are upregulated in ovarian tumors compared with associated normal tissues. (**A**) Volcano plot of ovarian tumor versus normal tissue proteomes. The fold changes in protein expression are plotted against the t-test p value (−log10). Red and green dots indicate the significance and fold change threshold, respectively (p-value < 0.05, fold change ≤ 0.8 or ≥1.3). HSP60 is highlighted. (**B**) Numbers of upregulated and downregulated proteins in mostly changed (with the largest absolute values of z-score and p-values < 0.05) canonical pathways according to ingenuity pathway analysis. Among these pathways, oxidative phosphorylation was the most activated (z-score = 6.325, −log(p-value) = 13.1) in ovarian tumors compared with the associated normal tissues. (**C**) Twenty-nine proteins related to oxidative phosphorylation were significantly upregulated in ovarian tumors. (**D**) Sixteen mitochondrial ribosomal proteins significantly increased in ovarian tumors. (**E**) Altered proteins associated with EIF2 and mTOR pathways. Data were analyzed by the Student’s t-test. p < 0.05 was considered as statistically significant. Error bars represent ± SEM.

Quantitative proteomic analysis was applied to investigate 10 pairs of ovarian tumor tissues and their associated normal tissue samples. We identified 7719 proteins, among which 5582 proteins were present in more than five pairs of samples. Missing values of these proteins were inputted in the Sequential KNN algorithm, and 2232 proteins were differentially expressed between tumor tissues and associated normal tissues. Among these proteins, 1925 were upregulated (abundance ratio ≥ 1.3, p-value ≤ 0.05), while 307 proteins were downregulated (abundance ratio ≤ 0.8, p-value ≤ 0.05) in ovarian tumors (Fig. 1A, Supplementary Tables S1 and S2). Ingenuity pathway analysis (IPA) identified 20 mostly altered pathways (with the largest absolute values of z-score and p-values < 0.05) that were related to tumor initiation and progression (Fig. 1B). Among these pathways, oxidative phosphorylation (OXPHOS), relying on five multimeric complexes which are embedded within the mitochondrial inner membrane^14^, had the largest z-score, which implied OXPHOS was most significantly activated (z-score = 6.325, −log(p-value) = 13.1) in tumor cells. Fatty acid β-oxidation I was also significantly activated in tumor cells. Other activated pathways included isoleucine degradation, gluconeogenesis, NAD phosphorylation and dephosphorylation, and NAD salvage pathway II. NAD biogenesis is essential for energy metabolism^15,16^. Among cellular growth and development signaling pathways, tRNA charging, eukaryotic initiation factor 2 (EIF2) signaling, and mTOR signaling were activated, indicating increased activities of protein translation in OC^17,18^. Other activated pathways included phosphoinositide metabolism, phospholipase C signaling, pyrimidine ribonucleotides biosynthesis, and PI3K signaling in OC. The NRF2-mediated stress response and mitochondrial L-carnitine shuttle pathway are detoxification pathways for effective clearance of ROS in OC^19,20^. The NF-κB pathway was activated in addition to remodeling of epithelial adherens junctions in the regulation of cell migration^21^.

OXPHOS was the most activated pathway in ovarian tumor cells compared with the non-tumor tissues, in which 29 proteins related to OXPHOS were significantly upregulated in OC, including 11 subunits of mitochondrial respiratory complex I, nine subunits of mitochondrial ATP synthase, six subunits of mitochondrial cytochrome c oxidase, CYC1, CYCS, and UQCRH (Fig. 1C). We also found upregulation of 16 mitochondrial ribosomal proteins including MRPL11, 17, 50, 1, 12, 44, 38, 15, 19, and 39, and MRPS27, 28, 31, 23, 22, and 33 (Fig. 1D). Proteins associated with EIF2 and mTOR pathways were also significantly upregulated in OC, including 8 subunits of eukaryotic translation initiation factor, 21 40S ribosomal proteins, DGKZ, PLD3, and PPP2R5C (Fig. 1E).

### Silencing or inhibition of HSP60 inhibits the proliferation of ovarian cancer cells

**Figure 2:**
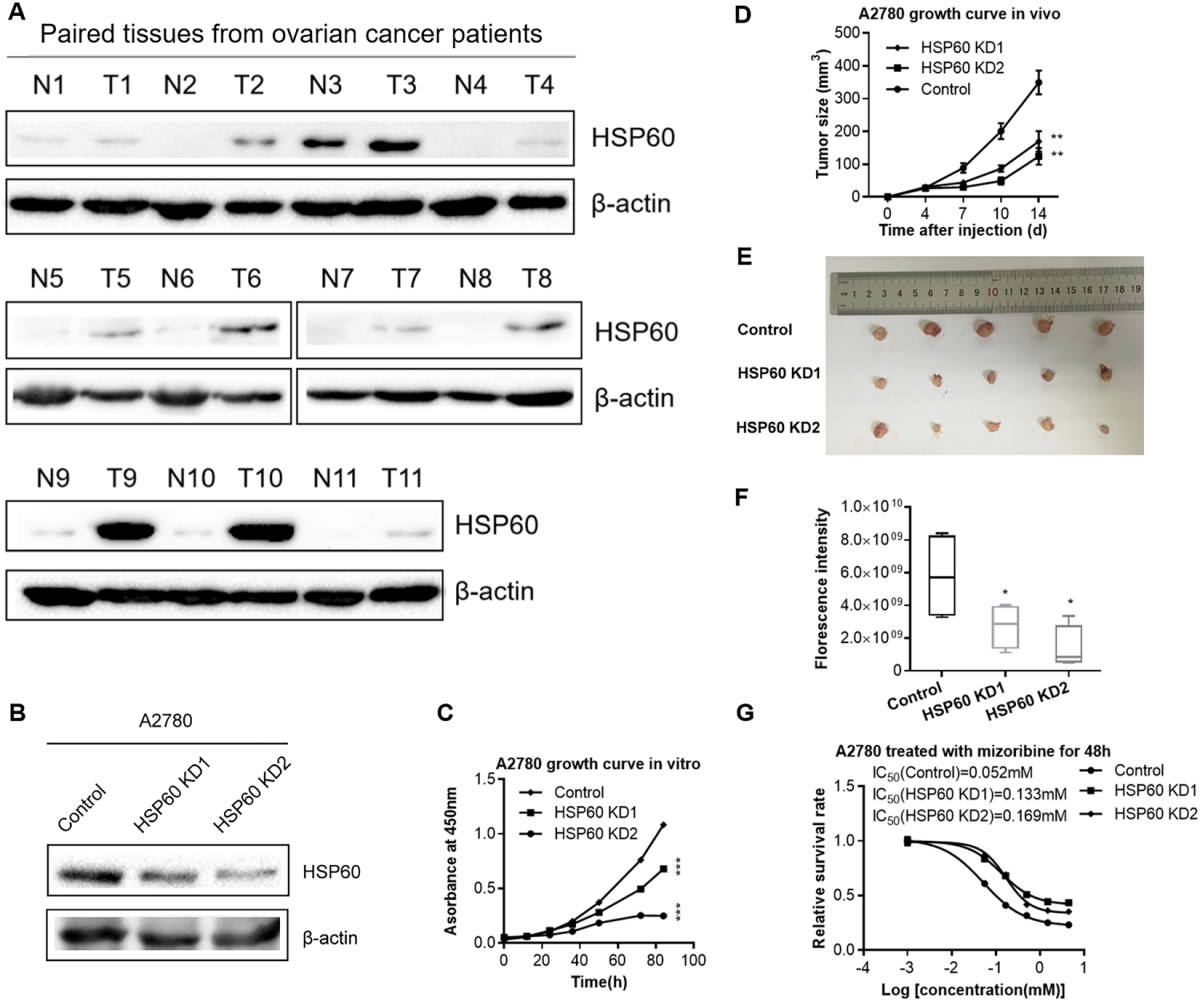
HSP60 knockdown decreases the growth rate of ovarian cancer cells *in vitro* and *in vivo*. (**A**) Western blotting images of HSP60 expression in 11 paired ovarian tumor tissues (T) and associated normal tissues (N). Juxtaposed images reflected western blotting results of samples gathered at different times. Multiple exposures with different exposure time are presented in Supplementary Fig. S7, full-length/uncropped gels and blots were presented in Supplementary Fig. S8. (**B**) Western blotting demonstrated that HSP60 was decreased in HSP60-KD A2780 cells compared with control cells. (**C**) Cell proliferation curve of HSP60-KD A2780 cells as compared to control cells. (**D**) Growth curves of tumors produced by HSP60-KD A2780 and control cells that were subcutaneously injected into mice. The volumes (mm^3^) of tumor were examined using digital calipers and estimated with the formula: π/6 × length (mm) × width (mm)^2^. (**E**) Images of tumors obtained from mice at 14 days after tumor cell injection. (**F**) Quantitation of fluorescence intensities. (**G**) Mizoribine attenuated the growth of HSP60-KD A2780 and control cells. P-values of the growth rate of Mizoribine-treated HSP60-KD1, KD2 A2780 and control cells were 0.0000037 and 0.00048, respectively. Data were calculated by the Student’s t-test. *p < 0.05, **p < 0.01, and ***p < 0.001. *p < 0.05 was considered as statistically significant. Error bars represent ± SEM. The bands of β-actin were from different part of the same gel with the corresponding bands of HSP60.

HSP60 is crucial for the maintenance of mitochondrial proteostasis. Proteomic results showed that HSP60 was one of the most upregulated proteins with the largest fold change and smallest p-value in tumor tissues compared with paired normal tissues (fold change = 2.7, p-value = 0.02) (Fig. 1A). This finding was confirmed by western blotting of 11 pairs of ovarian tumors and their paired normal ovarian tissues (Fig. 2A).

To examine the effects of HSP60 expression on tumor growth, we established stable A2780 cell line, in which HSP60 was knocked down by small hairpin RNA (shRNA) interference. The control cells were transfected with a scrambled shRNA without any homology in the human genome. Western blotting was used to analyze HSP60 silencing efficacy in A2780 cells, demonstrating that HSP60 expression was decreased by more than 50% in HSP60-KD1 cells and 70% in HSP60-KD2 cells (Fig. 2B). Further analysis by CCK-8 assays showed that HSP60 knockdown in A2780 cells resulted in a marked decrease in the proliferation rate (Fig. 2C). To examine the effects of HSP60 silencing on cell growth in a xenograft model, HSP60-KD1 and -KD2 cells were subcutaneously injected into immune-compromised mice. Fourteen days after injection, HSP60-KD-A2780 cells grew significantly slower than the control cells as quantified by a Vernier caliper, showing that the volumes of HSP60-KD1 and -KD2 tumors were 49% and 35% of the control tumors, respectively, and fluorescence intensity showing that HSP60-KD1 and -KD2 tumors were 47% and 24% of control tumors, respectively (Fig. 2D–F, Supplementary Fig. S1). The xenograft experiments revealed that HSP60 knockdown significantly slowed tumor growth. Furthermore, we found that HSP60 inhibition by mizoribine^22^ decreased the growth of A2780 and HSP60-KD A2780 cells (Fig. 2G). Importantly, A2780 cells were more susceptible to mizoribine treatment than HSP60-KD cells. These results demonstrated that high HSP60 expression is important to promote tumor progression of OC, which is a potential therapeutic target for OC treatment.

### HSP60 knockdown disrupts mitochondrial integrity and decreases protein synthesis

**Figure 3:**
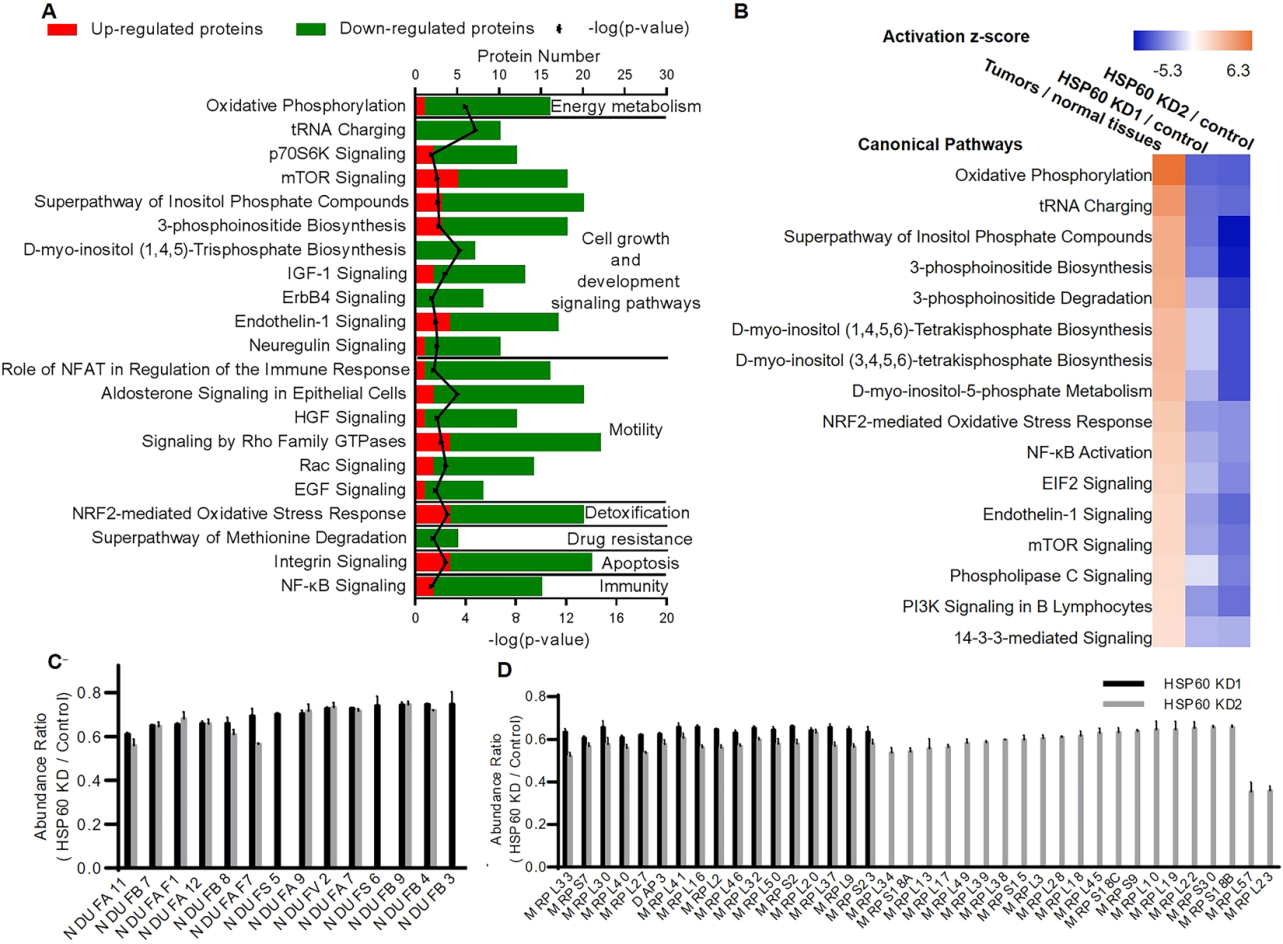
HSP60 knockdown disrupts mitochondria integrity and decreases protein synthesis. (**A**) Numbers of upregulated and downregulated proteins in mostly changed (with the largest absolute values of z-score and p-values < 0.05) canonical pathways according to ingenuity pathway analysis. Among these pathways, oxidative phosphorylation was the most inhibited (z-score = −3.5, p-value < 0.05) in HSP60-KD A2780 cells compared with control cells. (**B**) Comparative analysis of the z-scores of mostly changed pathways in clinical ovarian samples and HSP60-KD A2780 cells. (**C**) Fourteen and 11 subunits of mitochondrial complex I were downregulated in HSP60-KD1 and -KD2 A2780 cells, respectively. (**D**) Seventeen and 38 mitochondrial ribosomal proteins were downregulated in HSP60-KD1 and -KD2 A2780 cells, respectively. Data were analyzed by the Student’s t-test. p < 0.05 was considered as statistically significant. Error bars represent ± SEM.

To understand the mechanism of the effects of HSP60 knockdown on the growth of OC cells, quantitative proteomics was performed to identify differentially expressed proteins between control and HSP60-KD1 cells in biological quadruplicates, which identified 7683 proteins. Depending on the average reporter ion ratios in TMT quantitative analysis, 343 proteins were significantly downregulated (ratio < 0.67, p-value < 0.05), while 102 proteins were upregulated (ratio > 1.5, p-value < 0.05) in HSP60-KD1 cells compared with control cells (Supplementary Tables S3 and S4). The biological relevance of the differentially expressed proteins was analyzed by Gene Ontology with Panther (www.pantherdb.org/). Most differentially expressed proteins were associated with the cellular process and metabolic process (Supplementary Fig. S2). IPA showed that 21 cancer-related pathways were mostly inhibited (with the largest absolute values of z-score and p-values < 0.05) in HSP60-KD1 A2780 cells (Fig. 3A). Suppression of OSPHOS implied mitochondrial dysfunction in HSP60-KD A2780 cells, while decreased tRNA charging, p70S6K, and mTOR signaling pathways revealed that HSP60 silencing inhibited protein synthesis. Other pathways, including inositol phosphate and phosphoinositide metabolism, IGF-1 signaling, ErbB4 signaling, endothelin-1 signaling, and neuregulin signaling, were all inactivated in HSP60-KD cells. The NRF2-mediated oxidative stress response, integrin signaling, and NF-κB signaling were inactive in HSP60-KD cells, implying repressed detoxification of ROS, while apoptosis and cancer immunity^19,23,24^ were enhanced (Fig. 3A). More importantly, HSP60 silencing inactivated a series of pathways that were significantly activated in OC (Fig. 3B). Proteins identified and the results of IPA pathway analysis of HSP60-KD2 A2780 and control cells were shown in Supplementary Tables S3, S4 and Fig. S3, respectively.

Proteins associated with OXPHOS were upregulated in OC (Fig. 1C), but were downregulated in HSP60-KD cells. For example, 14 and 11 subunits of mitochondrial complex I were downregulated in HSP60-KD1 and -KD2 cells, respectively (Fig. 3C), and 17 and 38 mitochondrial ribosomal proteins were downregulated in HSP60-KD1 and -KD2 A2780 cells, respectively (Fig. 3D). These results indicated that HSP60 knockdown disrupted mitochondrial integrity and suppressed OXPHOS in A2780 cells. Furthermore, HSP60 knockdown upregulated expression of S-methyl-5′-thioadenosine phosphorylase (MTAP) and cytosolic purine 5′-nucleotidase (NT5C2) (Supplementary Tables S3 and S4). To further explore the effects of HSP60 silencing on protein synthesis and degradation, multiplexed proteome dynamics profiling (mPDP) was used to distinguish constitutive and synthesis dependent HSP60 clients. mPDP combines dynamic stable isotope labeling with amino acids in cell culture (SILAC) with isobaric mass tagging to analyze protein degradation and synthesis. Among the nascent proteins, HSP60 silencing downregulated (fold change < 0.8, p-value < 0.05) 41 mitochondrial ribosomal proteins and 9 complex I subunits in 2780 cells, while upregulated (fold change > 1.2, p-value < 0.05) 0 mitochondrial ribosomal proteins and 1 complex I subunit, indicating that HSP60 silencing decreased synthesis of these mitochondrial proteins. Moreover, HSP60 silencing downregulated 48 mitochondrial ribosomal proteins and 9 complex I subunits in the mature proteins, while upregulated neither mitochondrial ribosomal protein nor complex I subunit in the mature proteins, showing that HSP60 silencing also caused fast protein degradation (Supplementary Fig. S4, Tables S5 and S6). These results demonstrated that the levels of mitochondria ribosomal proteins and complex I subunits not only depend on HSP60 during protein synthesis, but their stability also consistently requires HSP60.

### Knockdown of HSP60 activates the adenine-AMPK pathway, which suppresses the mTOR pathway in ovarian cancer cells

**Figure 4:**
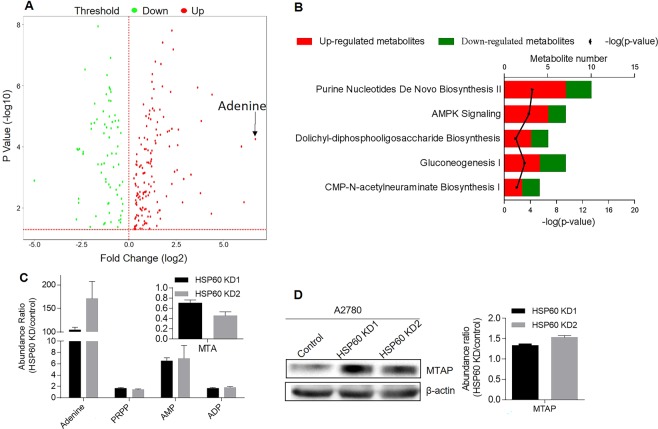
Metabolomic analysis of HSP60-KD A2780 and control cells. (**A**) Volcano plot of significantly changed metabolites in HSP60-KD1 A2780 and control cells. Expression fold changes are plotted against the t-test p value (−log10). Red and green dots indicate the significance and fold change threshold, respectively (p-value < 0.05, fold change ≤ 0.75 or ≥1.3). Adenine is highlighted. (**B**) Numbers of upregulated and downregulated metabolites in mostly changed (with the largest absolute values of z-score and p-values < 0.05) canonical pathways according to ingenuity pathway analysis. (**C**) Fold change of metabolites related to the adenine-AMPK pathway in HSP60-KD A2780 cells. (**D**) Western blotting and protein fold change of MTAP in HSP60-KD A2780 cells. Data were analyzed by the Student’s t-test. p < 0.05 was considered as statistically significant. Error bars represent ± SEM. The bands of β-actin were from different part of the same gel with the corresponding bands of MTAP.

The above results revealed that HSP60 knockdown reprogrammed cellular metabolism. Next, we performed metabolomics analysis to determine metabolite differences between HSP60-KD and control cells in five biological replicates. We found that HSP60 silencing increased adenine levels by 104-fold among 259 altered metabolites in HSP60-KD1 cells (Fig. 4A) and increased adenine by 171-fold among 232 altered metabolites in HSP60-KD2 cells (Supplementary Tables S7 and S8). IPA analysis of the significantly changed metabolites demonstrated that purine nucleotide de novo biosynthesis II and AMPK signaling pathways were the most activated (with the largest z-scores and p-values < 0.05) canonical pathways in HSP60 KD cells (Supplementary Tables S9 and S10). Other activated pathways included dolichyl-diphospho-oligosaccharide biosynthesis, gluconeogenesis, and CMP-N-acetylneuraminate biosynthesis I (Fig. 4B). We found that adenine precursor 5′-methylthioadenosine (MTA) was decreased by 30% and 64% in HSP60-KD1 and KD2 cells, respectively (Fig. 4C). Conversion of MTA to adenine is catalyzed by MTA phosphorylase (MTAP), which was upregulated in HSP60-KD A2780 cells based on proteomics and western blot results (Fig. 4D). MTAP-mediated MTA degradation is the main source of cellular adenine^25^. We also found that phosphoribosyl pyrophosphate (PRPP) was increased by 1.7- and 1.4-fold in HSP60-KD1 and -KD2 cells, respectively, while adenosine monophosphate (AMP), a product of the adenine phosphoribosyltransferase (APRT)-catalyzed reaction between adenine and PRPP^26^, was increased by 6.5- and 7-fold in HSP60-KD1 and -KD2 cells (Fig. 4C).

**Figure 5:**
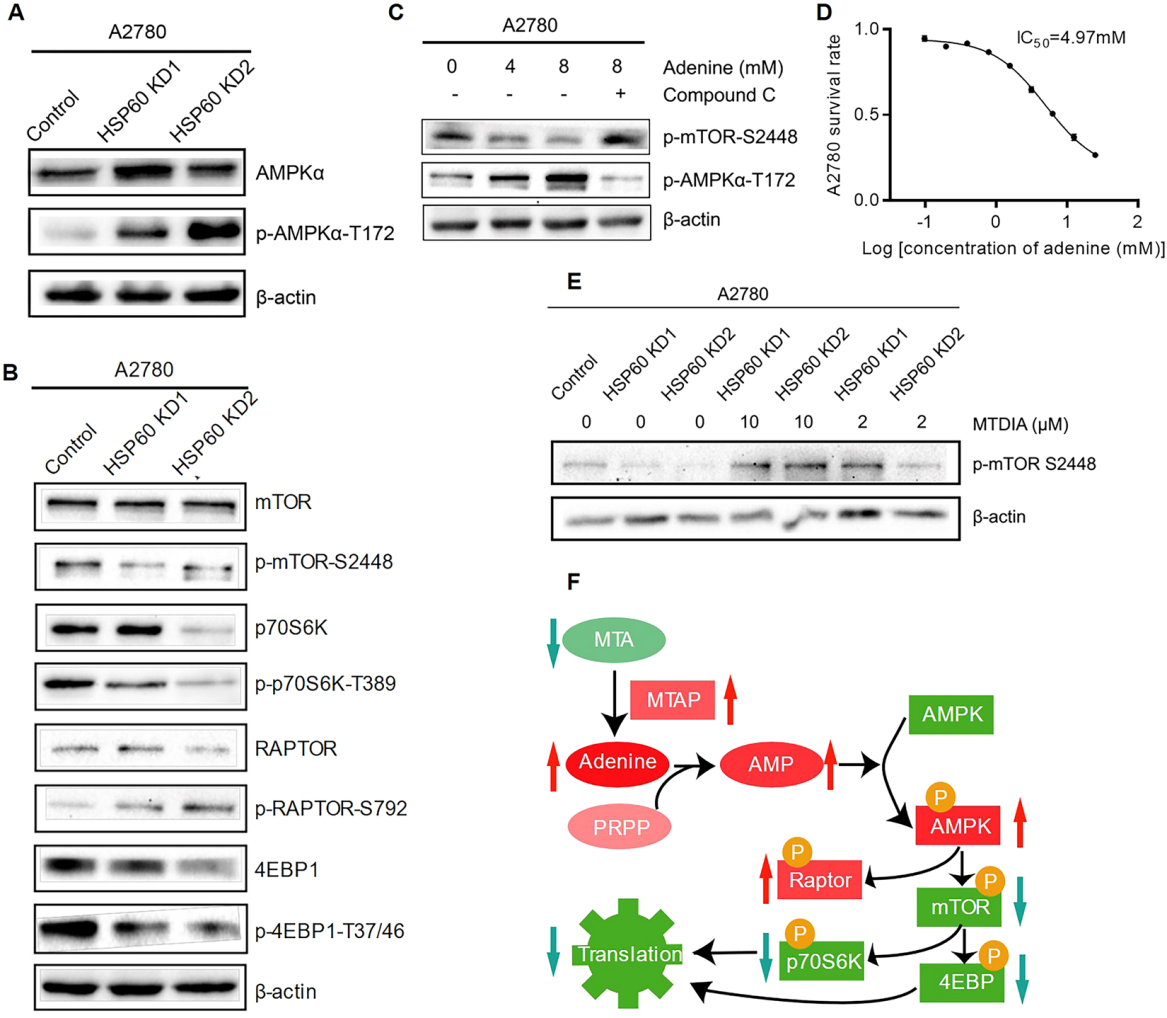
Knockdown of HSP60 activates the adenine-AMPK pathway and suppresses the mTOR pathway. (**A**) Increased phosphorylation of AMPK (T172) in HSP60-KD A2780 cells. (**B**) Inhibited mTOR pathway in HSP60-KD A2780 cells. (**C**) Increased p-AMPK and decreased p-mTOR in adenine-treated A2780 cells. p-AMPK and p-mTOR were restored in Compound C-treated HSP60-KD A2780 cells. (**D**) Cytotoxicity of adenine on A2780 cells. (**E**) MTDIA, as MTAP inhibitor, could rescue p-mTOR of HSP60-KD cells. (**F**) Conceptual representation of regulation of the adenine/AMPK/mTOR pathway. Error bars represent ± SEM.

Because AMP promotes AMPK signaling^27^, we hypothesized that the accumulated adenine increased AMP and consequently drove AMPK signaling in HSP60-KD cells. Western blotting showed that phosphorylation of AMPKα-T172 was significantly increased in HSP60-KD cells (Fig. 5A). The mTOR pathway is the known target of the activated AMPK in which raptor phosphorylation by AMPK prevents the formation of the mTORC1 complex^28^. Indeed, western blotting showed significantly increased phosphorylation of raptor, and decreased phosphorylation in S6K and 4EBP1 (Fig. 5B). Adenine treatment also increased AMPKα phosphorylation and inhibited mTOR phosphorylation, which was rescued by p-AMPK inhibitor dorsomorphin (Compound C) hydrochloride (Fig. 5C), indicating that accumulation of adenine in HSP60-KD cells could activate the AMPK pathway, thereby inhibiting the mTOR pathway. Furthermore, adenine treatment induced cell death of A2780 cells with an IC_50_ of 4.97 mM (Fig. 5D), indicating that adenine inhibited cell growth of OC. Then we treated HSP60-KD A2780 cells with MTAP inhibitor MT-DADMe-ImmA (MTDIA) and demonstrated that inhibition of production of adenine in cells by MTDIA could rescue the mTOR pathway of HSP60-KD1 and KD2 cells in a dose-dependent manner (Fig. 5E). The above results suggested that the activated adenine-AMPK pathway suppressed the mTOR pathway in HSP60-KD A2780 cells (Fig. 5F).

## Discussion

OC is one of the deadliest gynecological malignancies. Finding new therapeutic strategies is urgently needed to improve the 5-year survival rate that remains below 30%^4^. By analyzing paired ovarian tumor and normal ovarian tissues, we found that proteins associated with energy metabolism, cell growth, detoxification, immunity, and cell motility were significantly altered in OC, resulting in activation of two predominant cellular processes, OXPHOS and protein synthesis. The classic Warburg hypothesis proposes that dysfunctional mitochondria are the cause of the higher rate of glycolysis as well as the predominant cause of cancer development. The classic Warburg phenotypes were confirmed in some tumors, such as clear cell renal cell carcinoma^11^, but not in glioblastoma^12^. In ovarian cancer, proteins of OXPHOS were significantly upregulated in tumor samples, suggesting that the classical Warburg effects were not operative in this type of cancer. However, mitochondrial functions were essential for tumor progression.

HSP60 is the major chaperonin that regulates mitochondrial proteostasis. The roles of HSP60 in tumorigenesis and progression have been context dependent. The high expression of HSP60 were found in GBM, prostate cancer, and colon cancer, whereas HSP60 expressions are lower in ccRCC and bladder cancer as compared to the paracancerous tissues^10^. We analyzed data from The Cancer Genome Atlas (TCGA) and found that the expression levels of *HSPD1* mRNA were elevated in GBM, prostate cancer, colon cancer, and decreased in ccRCC (Supplementary Fig. S5). In our previous study, we detected that HSP60 expression was significantly lower in ccRCC tissue as compared to the adjacent paracancerous tissues^11^. HSP60-overexpression in ccRCC cells decreased cell growth, while knockdown of HSP60 enhanced glycolysis and decreased oxidative phosphorylation of ccRCC cells^29^.

In the present study, we proved that HSP60 was upregulated in OC tissues compared with normal tissues, and HSP60 knockdown reduced the proliferation of ovarian cancer cells. We performed proteomic analysis to identify differentially expressed proteins between HSP60-KD and control cells, revealing that HSP60 knockdown reversed most pathways activated in OC, and in particular, it significantly downregulated proteins associated with mitochondrial functions and protein synthesis. Furthermore, inhibition of HSP60 by mizoribine decreased cell growth and proliferation. These results indicated that HSP60 was a potential therapeutic target for ovarian cancer.

Our previous study showed that HSP60 silencing activated the AMPK pathway^11,12^. To explore the mechanisms underlying HSP60 silencing-mediated AMPK activation, we performed metabolomic analysis and surprisingly discovered that HSP60 silencing increased the cellular adenine level by more than 100-fold. Analysis of metabolomic data led us to hypothesize that MTAP-mediated degradation of MTA to adenine contributed to the increase of the cellular adenine level and activation of the AMPK pathway. We also found that MTAP was downregulated in seven ovarian tumor samples as compared with the paired normal tissues by western blotting (Supplementary Fig. S6), which correlated well with our results that HSP60 downregulation in ovarian cancer cells led to increase expression of MTAP. It has been reported that MTAP is downregulated in solid tumors and hematological malignancies because of *MTAP* deletion or methylation at the *MTAP* promoter region^30^. Downregulation of MTAP in OC has also been correlated with poor prognoses^31^. MTA is cleaved by MTAP to generate adenine and 5-methylthioribose-1-phosphate. The latter compound is a precursor of methionine^32^. In our metabolomic study, methionine was decreased significantly in HSP60-KD A2780 cells. S-adenosylmethionine (SAM) is the general methyl donor for methylation, and methionine is needed for the production of SAM^33^. We found that SAM was decreased in HSP60-KD A2780 cells, which might reduce methylation of the MTAP promoter, inducing MTAP expression.

Further analysis revealed that HSP60 silencing increased the AMP level by 8-fold, leading to the increased AMP/ATP ratio and AMPK activation. Indeed, HSP60 silencing significantly increased AMPK T172 phosphorylation. Previous studies have shown that activated AMPK inhibits formation of the mTORC1 complex via raptor phosphorylation. Our results showed that HSP60 knockdown activated AMPKα and inhibited mTOR and its downstream targets p70S6 kinase and 4EBP1 in A2780 cells. These results demonstrate that HSP60 silencing activates adenine/AMPK pathways to inhibit protein synthesis in OC cells (Fig. 5E). More importantly, we revealed that adenine treatment induced apoptosis of OC cells.

## Conclusion

Our results generated the proteome landscape of ovarian cancer and shed lights on understanding HSP60 functions in ovarian cancer progression. We demonstrated that HSP60 was essential in mitochondrial proteostasis. HSP60 knockdown interrupted the integrity of the respiratory chain and downregulated proteins involved in translation, led to accumulation of adenine and activation of the AMPK pathway, thereby inhibiting the mTOR pathway, which shut down protein synthesis and suppressed cell growth. Our data indicate that HSP60 is a potential target for ovarian cancer treatment.

## Materials and Methods

### Clinical human ovarian cancer samples

We collected 11 pairs of ovarian tumor tissue samples and associated normal ovarian tissue samples with written informed consent from patients with OC undergoing surgery at the Department of Gynecologic Oncology of Women’s Hospital of Zhejiang University School of Medicine (Hangzhou, China). Their clinical information was presented in Supplementary Table S11. Pathological features of tumors were confirmed according to post-operative pathology. Normal ovarian tissues were obtained from the contralateral ovaries that were confirmed normal via post-operative pathology. The study was approved by the Scientific and Ethics Committee of the Women’s Hospital of Zhejiang University School of Medicine. All experiments were performed in accordance with relevant guidelines and regulations. Informed consent was obtained from all participants and/or their legal guardians. Part of the specimen was stored at −80 °C. 8 M Urea in PBS (pH 7.4) was used to extract total proteins for proteomic study and western blotting.

### Quantitative proteomic analysis

Liquid chromatography – tandem mass spectrometry (LC–MS/MS) was used to carry out quantitative proteomic analysis as previously described^12,34^. Briefly, 100 μg proteins extracted from the clinical samples or cell lines were reduced by dithiothreitol and alkylated with iodoacetamide, followed by digestion with trypsin at 37 °C overnight. The purified peptides were labeled with TMT reagents (Thermo, Waltham, MA), mixed together, desalted and separated by reverse phase (RP) chromatography. The data-dependent acquisition method was used to acquire MS data by Orbitrap Q-Exactive mass spectrometer, utilizing Xcalibur 3.0 software. Proteomic analysis of cell lines was carried out in biological quadruplicates. The MS/MS spectra were cross-referenced against the UniProt human database, the search engine was SEQUEST from Proteome Discoverer Software (version 2.1).

### Ingenuity pathway analysis

Significantly changed proteins or metabolites (fold change < 0.8 or >1.3, p < 0.05) were uploaded into Ingenuity Pathway Analysis (IPA) software to perform pathway analysis. The p-values of pathways were calculated based on Fisher’s exact test right-tailed methods. The match between observed gene expression and expected relationship direction was measured with Z-score. Z-score > 2 is considered that pathway is significantly activated, z-score < −2 is considered that pathway is significantly inhibited, but a pathway does not have a good z-score does not make it uninteresting^35^.

### Western blotting

Western blot analysis followed a standard procedure. Cells were lysed in RIPA buffer (Solarbio). Protein concentrations were measured using the BCA protein assay kit. Equal amounts of proteins were run on a 12% SDS-PAGE gel and transferred onto a PVDF transfer membrane^11^. Anti-β-actin antibody was purchased from Abcolonal (Wuhan, China). Anti-HSP60, AMPKα, phospho-AMPKα (Thr172), Raptor, phosphor-RAPTOR (Ser792), 4EBP, and phosphor-4EBP (Thr37/46) antibodies were obtained from Cell Signaling Technology (Danvers, MA). mTOR, phosphor-mTOR (Ser2448), p70s6k, phosphor-p70S6K (Thr389) antibodies were obtained from Sigma (St Louis, MO). MTAP antibody was obtained from Proteintech (Chicago, IL). The Immunoblots were detected using ECL reagents (Engreen, China) and Chemidoc Apparatus with Image Lab software.

### Cell lines

Human ovarian cancer A2780 cell line (A2780) was purchased from the Cell Bank of Type Culture Collection of Chinese Academy of Sciences (Shanghai, China). A2780 cells were grown in DMEM media (Wisent, Montreal, QC). The culture media was supplemented with 10% fetal bovine serum (Wisent, Montreal, QC) and 1% penicillin/streptomycin (Wisent, Montreal, QC)^11^.

### Establishment of stable HSP60 knockdown cell lines

Production of lentiviral particles of HSP60-shRNAs was performed according to the protocol published by Tiscornia *et al*.^36^. The two shRNAs targeting HSP60 were chosen based on previous study and were confirmed not targeting homologous sequences by NCBI BLAST^37^. A scrambled non-silencing shRNA without homologous sequence in the human genome was used as negative control^12^. The shRNA sequences were listed in Supplementary Table S12. The annealing conditions were as follows: incubation at 95 °C for 2 min, decreased to 25 °C by 0.1 °C/s, and 4 °C for 10 min.

The products were added phosphate group with ATP and T4 PNK (NEB) and purified with gel DNA purification kit (TIANGEN). Restriction sites were created on the double strand shRNAs and pll3.7 vector, and the shRNAs were inserted into plasmid pLL3.7 lentivirus vectors respectively with T4 DNA ligase. The ligation products were transformed to competent DH5α cells (TIANGEN)^38^. After incubating the plates at 37 °C overnight, we validated five colonies randomly for each transformation by DNA sequencing. The correct plasmids were isolated with DNA extraction kit (TIANGEN).

Pll3.7-shRNAs were co-transfected with p MD2.G, p MDLg/p RRE and p REV-Rev into 293 T cells using PEI when cells reached 60–70% confluence. 72 h later, supernatants were harvested with PEG6000 and were used to infect A2780 cells with 6 μg/ml of polybrene when cells reached 30–40% confluence. GFP positive monoclonal cells were sorted into one single well of a 96-well plate by flow cytometer and cultured to generate stable cell lines, the expression level of HSP60 was measured by western blotting. Two HSP60-silencing cell lines HSP60-KD1 and HSP60-KD2 were established with two different shRNAs targeting HSP60. The control cell line was established with the scrambled non-silencing shRNA.

### Detection of cell proliferation by CCK-8 assay

Cells were digested and seeded into 96-well plates (100 μL/well; 2000 cells/well) and 5 wells were included in each group. Cells were incubated at 37 °C in an environment with 5% CO_2_. At 0, 12, 24, 36, 48, 72, and 84 h, 10 μL of CCK-8 solution (Dojindo Co., Ltd. Japan) was added into each well, followed by incubation for 1.5 h, then absorbance was measured at 450 nm.

For detecting survival rate of A2780 cells treated with mizoribine or adenine, cells were seeded into 96-well plates (100 μL/well; 5000 cells/well), 5 wells were included in each group. After culture of 6 h, cells were treated with mizoribine (4.5, 1.5, 0.5, 0.17, 0.06, 0 mM, for 48 h) or adenine (25, 12.5, 6.25…0 mM, for 24 h). The CCK-8 reagent was added to treated cells and cultured at 37 °C for 1.5 h. A microplate reader was used to measure optical density (OD) at 450 nm^39^.

### Xenograft experiments

All animal studies were approved by the Animal Research Ethics Committee of the Tsinghua University. All experiments were performed in accordance with relevant guidelines and regulations. For xenograft experiments, 6 × 10^6^ HSP60-KD1, HSP60-KD2 A2780 and the control cells were harvested, washed, resuspended in 150 μL PBS and injected subcutaneously into 5-week-old nude mice (Vital River Company, China). Tumor sizes at 14th days after injection were quantified via fluorescence imaging using IVIS *in vivo* imaging system (Perkin Elmer, Waltham, MA)^11^. At the end of imaging, tumor samples from the three animal groups were harvested.

### Multiplexed proteome dynamics profiling

Multiplexed proteome dynamics profiling (mPDP) was used to analyze protein degradation and synthesis, it combined mass-spectrometry-based method and dynamic-SILAC labeling with isobaric mass tagging^40^. The experiment was carried out in biological triplicates. A2780 cells were grown in light SILAC medium containing arginine ^12^C_6_^14^N_4_ and lysine ^12^C_6_^14^N_2_, the shRNA targeting HSP60 and control shRNA were transfected transiently into A2780 cells respectively, and cultured for 48 h, then the medium was changed to the heavy SILAC medium containing stable isotope-enriched amino acids arginine ^13^C_6_^15^N_4_ and lysine ^13^C_6_^14^N_2_, after 6 h of incubation, cells were lysed, and proteins were digested with trypsin, and labeled with TMT reagents. After that, all samples were mixed and subjected to LC-MS/MS. The abundance of mature peptides and newly synthesized peptides could be measured using reporter ions in tandem mass spectra of SILAC light and heavy-encoded peptide ion signals.

### Metabolomic analysis

Metabolomic analysis was carried out as previously described^11^. Briefly, the extracted metabolites were concentrated in 80% methanol and subjected to LC-MS/MS analysis. The Q-Exactive Mass Spectrometer was used to carry out untargeted metabolomic profiling. To perform targeted quantitative analysis, we used the TSQ Quantiva™ Triple Quadrupole Mass Spectrometer with positive/negative ion switching. Retention time on the LC analysis and TraceFinder were used to identify metabolites.

### Statistical analysis

For proteomic analysis of 10 pairs of clinical samples, proteins with the missing value (whose peak intensities was 0 s) ratio above 50% were removed and proteins with missing value rates less than 50% were remained, missing values of these proteins were imputed with the Sequential KNN algorithm^41,42^. Proteomic analysis was performed using four biological replicates, multiplexed proteome dynamics profiling was carried out in biological triplicates. Metabolomic analysis was carried out in five biological replicates. Significantly changed proteins and metabolites were determined using the two-tailed Student’s t test and screened by volcano plot analysis using R (V.3.3.2). Xenograft experiments and detection of cell survival rates were carried out at least in three biological or technical replicates. Two-sided unpaired t tests were carried out with GraphPad Prism 6.0 software. P values of <0.05 were considered significant.

## Supplementary information

Supplementary Figures

Supplementary Datasets

## Data Availability

The mass spectrometry proteomics data have been deposited to the ProteomeXchange Consortium via the PRoteomics IDEntifications (PRIDE)^43^ partner repository with the dataset identifier PXD013811.

## References

[1] Bray F (2018). Global cancer statistics 2018: GLOBOCAN estimates of incidence and mortality worldwide for 36 cancers in 185 countries. CA: a cancer journal for clinicians.

[2] Hartnett J, Thom B, Kline N (2016). Caregiver Burden in End-Stage Ovarian Cancer. Clinical journal of oncology nursing.

[3] Pignata S (2011). Chemotherapy in epithelial ovarian cancer. Cancer Lett.

[4] Chu CS, Kim SH, June CH, Coukos G (2008). Immunotherapy opportunities in ovarian cancer. Expert review of anticancer therapy.

[5] Zhang H (2016). Integrated Proteogenomic Characterization of Human High-Grade Serous Ovarian Cancer. Cell.

[6] Coscia F (2018). Multi-level Proteomics Identifies CT45 as a Chemosensitivity Mediator and Immunotherapy Target in Ovarian Cancer. Cell.

[7] Arya R, Mallik M, Lakhotia SC (2007). Heat shock genes - integrating cell survival and death. Journal of biosciences.

[8] Ghosh JC, Siegelin MD, Dohi T, Altieri DC (2010). Heat shock protein 60 regulation of the mitochondrial permeability transition pore in tumor cells. Cancer research.

[9] Xanthoudakis S (1999). Hsp60 accelerates the maturation of pro-caspase-3 by upstream activator proteases during apoptosis. The EMBO journal.

[10] Cappello F, Conway de Macario E, Marasa L, Zummo G, Macario AJ (2008). Hsp60 expression, new locations, functions and perspectives for cancer diagnosis and therapy. Cancer biology & therapy.

[11] Tang H (2016). Downregulation of HSP60 disrupts mitochondrial proteostasis to promote tumorigenesis and progression in clear cell renal cell carcinoma. Oncotarget.

[12] Tang H (2016). Down-regulation of HSP60 Suppresses the Proliferation of Glioblastoma Cells via the ROS/AMPK/mTOR Pathway. Scientific reports.

[13] Hjerpe E (2013). HSP60 predicts survival in advanced serous ovarian cancer. International journal of gynecological cancer: official journal of the International Gynecological Cancer Society.

[14] Barrientos, A., Fontanesi, F. & Diaz, F. Evaluation of the mitochondrial respiratory chain and oxidative phosphorylation system using polarography and spectrophotometric enzyme assays. *Current protocols in human genetics* Chapter 19, Unit19.13, 10.1002/0471142905.hg1903s63 (2009). 10.1002/0471142905.hg1903s63 PMC2771113 19806590

[15] Yang Y, Sauve AA (2016). NAD(+) metabolism: Bioenergetics, signaling and manipulation for therapy. Biochimica et biophysica acta.

[16] Houtkooper RH, Canto C, Wanders RJ, Auwerx J (2010). The secret life of NAD+: an old metabolite controlling new metabolic signaling pathways. Endocrine reviews.

[17] Kimball SR (1999). Eukaryotic initiation factor eIF2. The international journal of biochemistry & cell biology.

[18] Saxton RA, Sabatini DM (2017). mTOR Signaling in Growth, Metabolism, and Disease. Cell.

[19] Kovac S (2015). Nrf2 regulates ROS production by mitochondria and NADPH oxidase. Biochimica et biophysica acta.

[20] Ye J (2010). L-carnitine attenuates oxidant injury in HK-2 cells via ROS-mitochondria pathway. Regulatory peptides.

[21] Le Bras GF, Taubenslag KJ, Andl CD (2012). The regulation of cell-cell adhesion during epithelial-mesenchymal transition, motility and tumor progression. Cell adhesion & migration.

[22] Itoh H, Komatsuda A, Wakui H, Miura AB, Tashima Y (1999). Mammalian HSP60 is a major target for an immunosuppressant mizoribine. The Journal of biological chemistry.

[23] Xia Y, Shen S, Verma IM (2014). NF-kappaB, an active player in human cancers. Cancer immunology research.

[24] Aoudjit F, Vuori K (2012). Integrin signaling in cancer cell survival and chemoresistance. Chemotherapy research and practice.

[25] Kaneko K, Fujimori S, Kumakawa T, Kamatani N, Akaoka I (1991). Disturbance in the metabolism of 5’-methylthioadenosine and adenine in patients with neoplastic diseases, and in those with a deficiency in adenine phosphoribosyltransferase. Metabolism: clinical and experimental.

[26] Mills GC, Schmalstieg FC, Trimmer KB, Goldman AS, Goldblum RM (1976). Purine metabolism in adenosine deaminase deficiency. Proc Natl Acad Sci USA.

[27] Jeon SM (2016). Regulation and function of AMPK in physiology and diseases. Exp Mol Med.

[28] Wang Z, Wang N, Liu P, Xie X (2016). AMPK and Cancer. Experientia supplementum (2012).

[29] Teng R (2019). HSP60 silencing promotes Warburg-like phenotypes and switches the mitochondrial function from ATP production to biosynthesis in ccRCC cells. Redox Biol.

[30] Bosio M (2018). 5-hydroxymethylcytosine but not MTAP methylation status can stratify malignant pleural mesothelioma based on the lineage of origin. Multidisciplinary respiratory medicine.

[31] Ding N (2017). MTAP deficiency is associated with an unfavourable prognosis and platinum resistance in ovarian cancer. Int J Clin Exp Pathol.

[32] Bertino JR, Waud WR, Parker WB, Lubin M (2011). Targeting tumors that lack methylthioadenosine phosphorylase (MTAP) activity: current strategies. Cancer biology & therapy.

[33] Glier MB, Green TJ, Devlin AM (2014). Methyl nutrients, DNA methylation, and cardiovascular disease. Mol Nutr Food Res.

[34] Gu L (2015). Functional Characterization of Sirtuin-like Protein in Mycobacterium smegmatis. J Proteome Res.

[35] Kramer A, Green J, Pollard J, Tugendreich S (2014). Causal analysis approaches in Ingenuity Pathway Analysis. Bioinformatics.

[36] Tiscornia G, Singer O, Verma IM (2006). Production and purification of lentiviral vectors. Nat Protoc.

[37] Tiedemann RE (2012). Identification of molecular vulnerabilities in human multiple myeloma cells by RNA interference lethality screening of the druggable genome. Cancer research.

[38] Zeng F (2017). AFEAP cloning: a precise and efficient method for large DNA sequence assembly. BMC Biotechnol.

[39] Wang W (2018). Decreased NAD Activates STAT3 and Integrin Pathways to Drive Epithelial-Mesenchymal Transition. Mol Cell Proteomics.

[40] Savitski MM (2018). Multiplexed Proteome Dynamics Profiling Reveals Mechanisms Controlling Protein Homeostasis. Cell.

[41] Wang S (2018). MetaboGroup S: A Group Entropy-Based Web Platform for Evaluating Normalization Methods in Blood Metabolomics Data from Maintenance Hemodialysis Patients. Anal Chem.

[42] Mader P, Gotel O (2012). Towards automated traceability maintenance. J Syst Softw.

[43] Perez-Riverol Y (2019). The PRIDE database and related tools and resources in 2019: improving support for quantification data. Nucleic Acids Res.

