# Supplementary material for: HSP60-regulated Mitochondrial Proteostasis and Protein Translation Promote Tumor Growth of Ovarian Cancer
Source: Sci Rep. 2019 Sep 2;9:12628. doi: 10.1038/s41598-019-48992-7 (PMC6718431; doi:10.1038/s41598-019-48992-7)
Supplement: Supplementary file 1 — Supplementary Figures [file 41598_2019_48992_MOESM1_ESM.docx]

**HSP60-regulated Mitochondrial Proteostasis and Protein Translation** **Promote** **Tumor Growth of Ovarian Cancer**

Jianying Guo^1^, Xiao Li^2^, Wenhao Zhang^1^, Yuling Chen^1^, Songbiao Zhu^1^, Liang Chen^5^, Renhua Xu^3^, Yang Lv^4^, Di Wu^4^, Mingzhou Guo^4^, Xiaohui Liu^1^, Weiguo Lu^2*^, Haiteng Deng^1*^


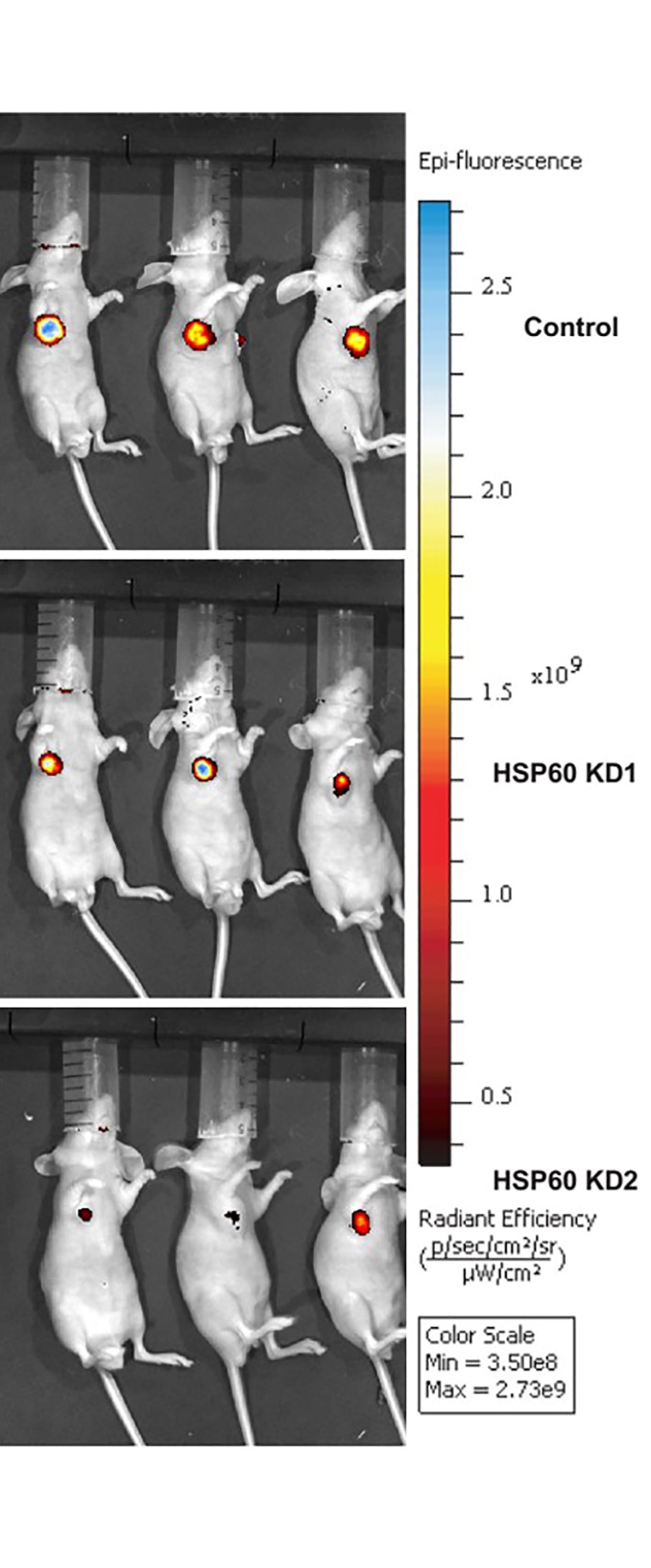


**Supplementary Figure S1. Florescent imaging of xenografts. HSP60-KD-A2780 cells grew significantly slower than the control cells.**


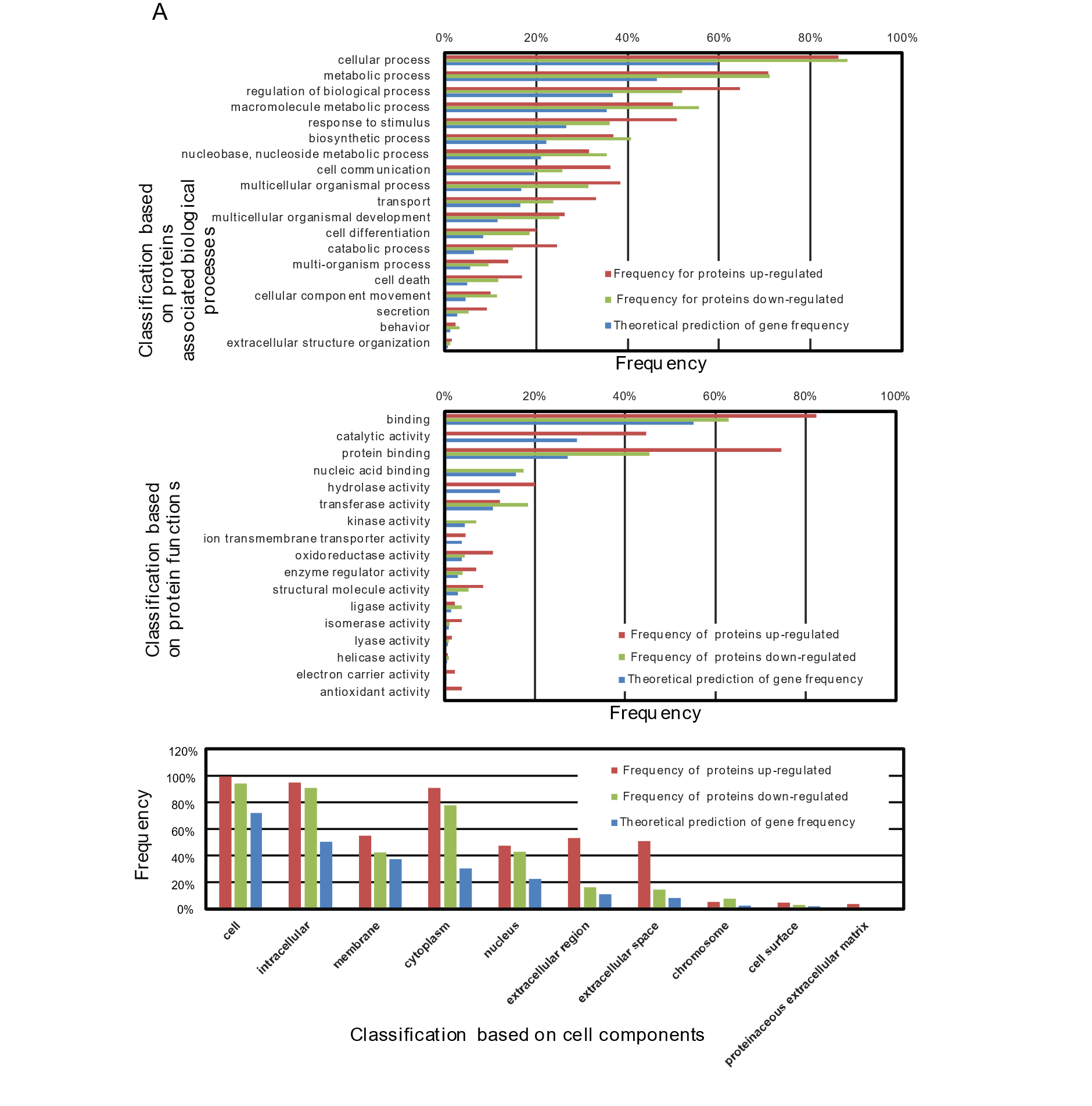
 **Supplementary Figure S2.** **Gene Ontology analysis of differentially expressed proteins in HSP60-KD and control A2780 cells.**

**Supplementary Figure S3. IPA pathway analysis of HSP60-KD2 A2780 and control cells.**

Numbers of upregulated and downregulated proteins in mostly changed (with the largest absolute values of z-score and p-values < 0.05) canonical pathways according to IPA. Oxidative phosphorylation was one of the most inhibited pathways (z-scores < -2, p-value < 0.05) in HSP60-KD2 A2780 cells compared with control cells.

**Supplementary** **F****igure S4. Multiplexed proteome dynamics profiling (mPDP) of mitochondrial proteins that were constitutive and synthesis dependent on HSP60.**

The fold change of mature (light) and nascent (heavy) mitochondrial ribosomal proteins and subunits of complex I in HSP60-KD A2780 cells compared with control cells was shown.


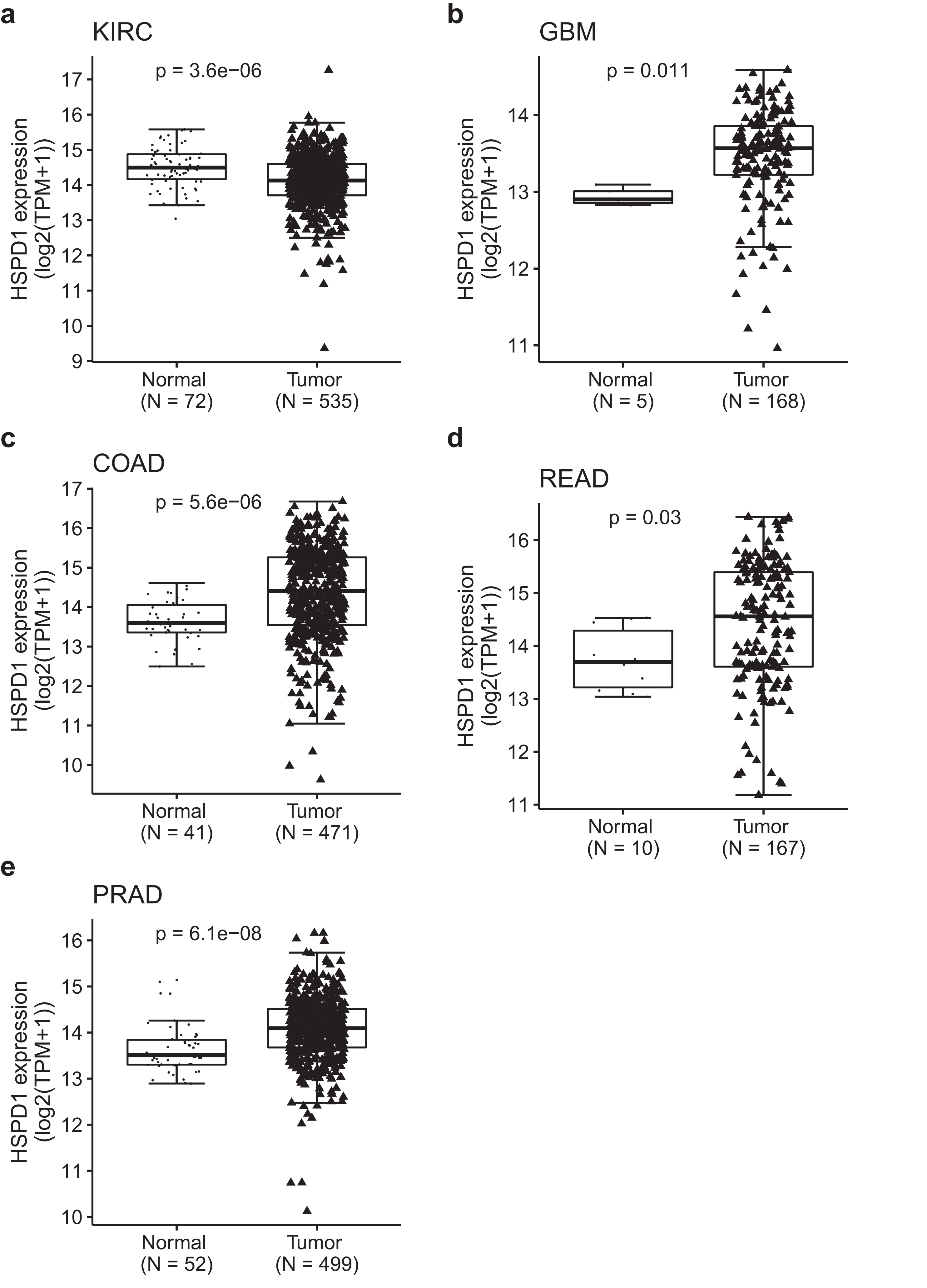


**Supplementary Figure S5. Expression levels of *HSPD1* mRNA in different cancer tissues as compared to the normal tissues. (a)** kidney renal clear cell carcinoma (KIRC). **(b)** glioblastoma (GBM). **(c)** colon adenocarcinoma (COAD). **(d)** rectum adenocarcinoma (READ). **(e)** prostate adenocarcinoma (PRAD). Data were analyzed by the Wilcoxon method. p<0.05 was considered as statistically significant. Error bars represent ± SEM.

**
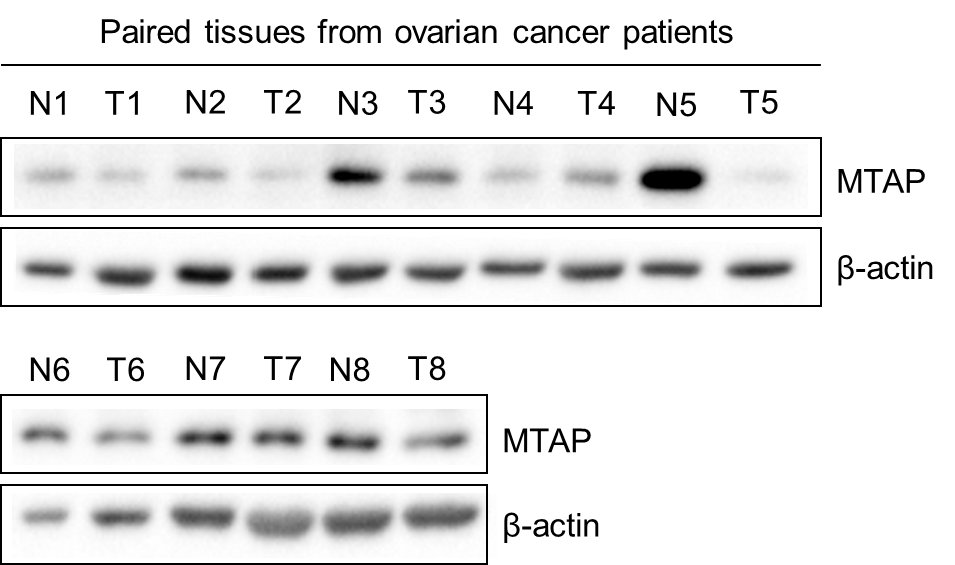
**

**Supplementary Figure S6. Western blotting of the expression levels of HSP60 in paired ovarian tumor tissues (T) and associated normal tissues (N).**


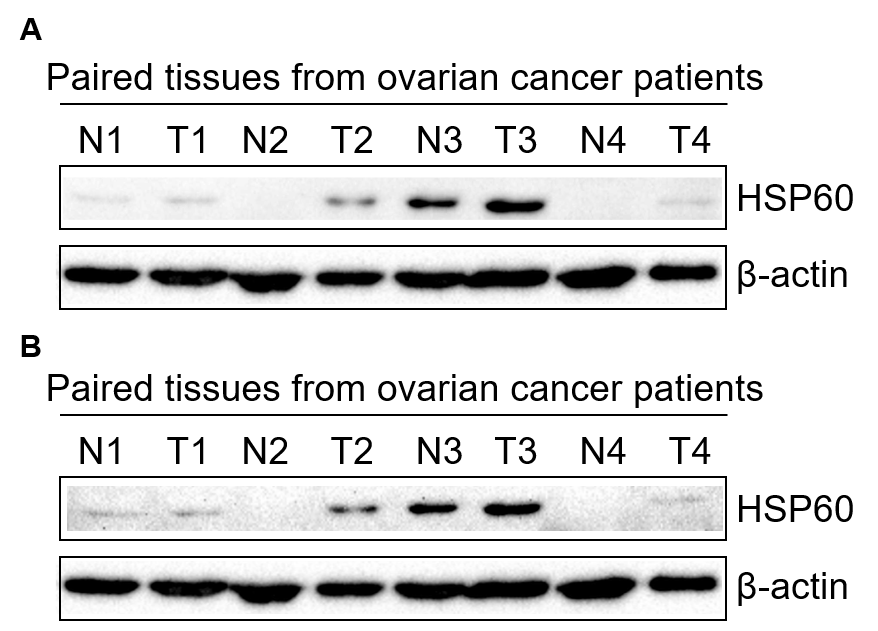


**Supplementary Figure S7.** **Multiple exposures of Western blotting of the expression levels of HSP60 in 4 paired ovarian tumor tissues (T) and associated normal tissues (N),** **with exposure time of (A) 80 seconds and (B) 50 seconds.**


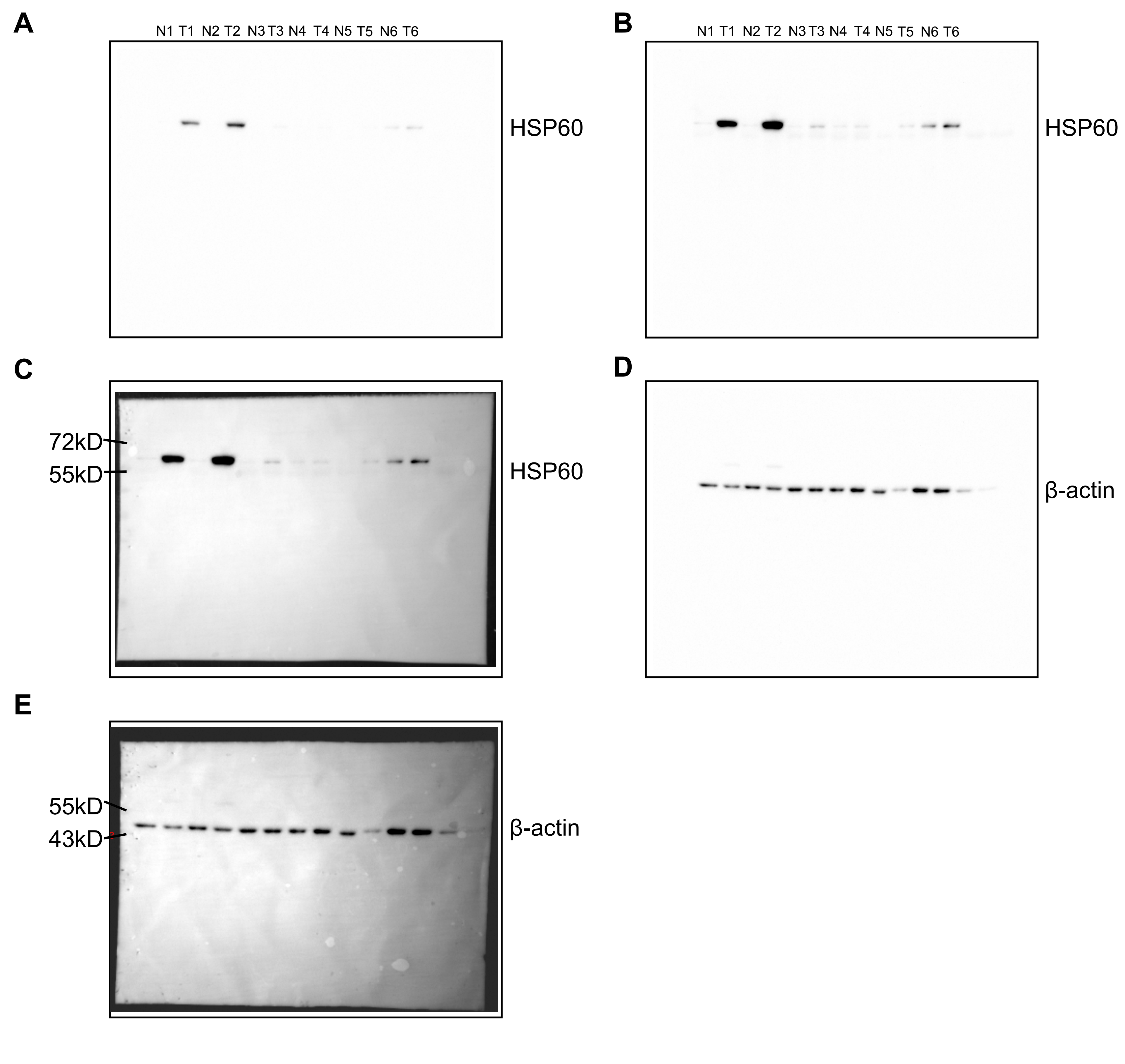


**Supplementary Figure S8. Full-length/uncropped gels and blots of HSP60 expression in paired ovarian tumor tissues (T) and normal tissues (N).**

Multiple exposures of Western blotting of HSP60 in the OC specimens with exposure time of **(A)** 2 seconds and **(B)** 12 seconds, and **(C)** incorporated image with markers. **(D)** Western blotting of associated β-actin and **(E)** incorporated image with markers
